# Socioeconomic status and central adiposity as determinants of stress-related biological responses relevant to cardiovascular disease risk

**DOI:** 10.1016/j.bbi.2018.11.019

**Published:** 2019-03

**Authors:** Andrew Steptoe, Tanja-Julia Hiltl, Jennifer Beam Dowd, Mark Hamer

**Affiliations:** a Department of Behavioural Science and Health, University College London, London WC1E 6BT, UK; b Department of Global Health and Social Medicine, King’s College London, London WC2R 2LS, UK; c School of Sport, Exercise and Health Sciences, Loughborough University, Leicestershire LE11 3TU, UK

**Keywords:** Stress reactivity, Interleukin 6, Obesity, Cytomegalovirus, Cortisol, Blood pressure

## Abstract

Stress-related processes have been implicated in the associations between lower socioeconomic status (SES), central adiposity, and cardiovascular disease risk. This study analysed the impact of SES and central adiposity on cardiovascular, inflammatory and neuroendocrine stress responses, and associations with cytomegalovirus (CMV) infection in a sample of 537 men and women aged 53–76 years (mean 62.89 years). SES was defined by grade of employment (higher, intermediate, and lower categories), and central adiposity was indexed by waist-hip ratio (WHR). Cardiovascular, inflammatory and cortisol responses were monitored during administration of a standardized mental stress testing protocol and salivary cortisol was measured repeatedly over the day. Lower SES was associated with raised systolic and diastolic blood pressure (BP), plasma interleukin (IL-6), fibrinogen, C-reactive protein, and salivary cortisol, and a large WHR accentuated SES differences in fibrinogen, C-reactive protein, and likelihood of CMV seropositivity, independently of general adiposity indexed by body mass index. During mental stress testing, return to resting levels (recovery) following behavioural challenge in systolic and diastolic BP and heart rate was impaired among lower SES participants, particularly those with large WHR. Lower SES participants had greater cortisol concentrations across the day, but this pattern did not vary with WHR. These findings extend the evidence relating lower SES to stress-related biological risk factors for cardiovascular disease, and indicate that central adiposity may augment these effects.

## Highlights

• Lower SES is associated with greater inflammation and impaired blood pressure recovery.
• SES differences in inflammation are accentuated by central adiposity.
• Low SES people with central adiposity have higher rates of cytomegalovirus infection.
• Cortisol output over the day is inversely related to SES.
• These processes potential link SES and adiposity with cardiovascular disease risk.

## 1 Introduction

There are marked socioeconomic disparities in cardiovascular disease (CVD), with greater incidence of coronary heart disease (CHD), hypertension and stroke in lower socioeconomic status (SES) groups defined by occupation, education, income or self-defined social status (Kerr et al., 2011, Leng et al., 2015, Manrique-Garcia et al., 2011, Tang et al., 2016). Lifestyle factors such as smoking and sedentary behaviour make an important contribution to SES differences (Stringhini et al., 2011), but direct influences on biological processes implicated in CVD are also relevant (Kivimaki and Steptoe, 2018). Psychological stress elicits disturbances in autonomic regulation, neuroendocrine factors, inflammatory markers and endothelial function (Boylan et al., 2018, Marsland et al., 2017, Wirtz and von Kanel, 2017). Studies of acute mental stress have shown that lower SES is associated with delayed recovery (return towards baseline after stress) in systolic and diastolic blood pressure (BP) and in haemostatic variables, together with elevated concentration of markers of inflammation such as interleukin 6 (IL-6) (Boylan et al., 2018, Brydon et al., 2004). These responses are markers of chronic allostatic load (McEwen and Gianaros, 2010), and predict future cardiovascular morbidity (Panaite et al., 2015, Steptoe et al., 2016).

Central or visceral adiposity is a growing health problem, and is a pivotal risk factor for cardiometabolic disease (Balkau et al., 2007, Despres, 2012). Fat deposits in the abdominal visceral region are highly active in terms of autonomic, immune and neuroendocrine regulation, with inflammation, sympathetic nervous system activation, and disturbances of hypothalamic-pituitary-adrenocortical (HPA) function being implicated in central adiposity (Bjorntorp, 2001, Mancia et al., 2007, Speaker and Fleshner, 2012). Psychological stress may contribute to the development of central adiposity through alterations in stress biology (Dallman et al., 2005, Tomiyama, 2018). A positive association between central adiposity, inflammatory cytokine responses, and delayed BP recovery following acute mental stress has been described (Brydon et al., 2008, McInnis et al., 2014, Steptoe and Wardle, 2005), although an inverse association with heart rate and cortisol responses to stress has also been noted (Jones et al., 2012, Phillips et al., 2012).

Since central adiposity is more common among lower than higher SES groups, it is possible that the two share common pathways to cardiovascular risk through disturbed stress biology. However, the combined effect of lower SES and central adiposity has not previously been examined. We therefore investigated the impact of SES and central adiposity on inflammatory, hemodynamic and neuroendocrine responses to psychological stress in a sample of healthy middle-aged and older men and women drawn from the Whitehall II epidemiologic cohort. We hypothesized that lower SES and greater central adiposity would be associated with delayed cardiovascular (BP and heart rate) recovery following acute stress, with greater inflammatory responses to stress, greater cortisol output over the day, and with higher baseline levels. We further conjectured that these two factors might be synergistic, with the greatest responses emerging among lower SES individuals with greater central adiposity. Because the focus of this study was on central adiposity, body mass index (BMI) was included as a covariate in the analyses to take account of overall adiposity.

We also evaluated associations between SES and central adiposity and cytomegalovirus (CMV) serostatus. Herpesviruses such as CMV are frequently acquired early in life, and are more common among lower SES individuals as defined by education, poverty or parental occupational status (Dowd et al., 2012, Janicki-Deverts et al., 2014, Simanek et al., 2009). Positive CMV serostatus has been shown to predict future cardiovascular disease (Hui et al., 2016, Ji et al., 2012), and is implicated in inflammatory diseases and in the dysregulation of the hypothalamic-pituitary-adrenocortical (HPA) axis as well. We have previously found that CMV is associated with a flatter cortisol rhythm over the day, and with accelerated telomere shortening over three years (Dowd et al., 2017, Steptoe et al., 2009). CMV also affects metabolic processes, though associations with adiposity are poorly understood (Fleck-Derderian et al., 2017). Associations between CMV seropositivity, SES and central adiposity were therefore assessed in this study.

## 2 Methods

### 2.1 Participants

We recruited a sample of 543 healthy white European men (n = 294) and women (n = 249) aged 53–76 years (M = 62.89 ± 5.65) from the Whitehall II epidemiological cohort of British civil servants (Marmot et al., 1991). To be included in the study the participants were required to have no history of CHD, hypertension, inflammatory conditions or allergies. These criteria were used because the overarching aim of the study was to investigate psychosocial and biological risk factors for CHD (Hackett et al., 2012, Steptoe et al., 2010). Since one of the primary purposes of the study was to compare SES groups, we used a stratified sampling method to recruit potential participants from higher, intermediate, and lower grade of employment categories in the Whitehall II cohort. Because of the differences in cardiovascular disease across the socioeconomic gradient, there were fewer lower than higher or intermediate grade disease-free individuals eligible for the study, so the lower grade group was smaller. Participants were instructed not to take any anti-inflammatory or anti-histamine medication in the seven days prior to the testing day and to refrain from drinking alcohol and excessive exercise from the previous evening. The participants were also required to avoid caffeinated beverages and smoking at least 2 h prior to the testing session. Individuals presenting with colds or infections on the testing day were rescheduled. Civil service employment grade was used to divide participants into low, intermediate and high SES groups. Waist-hip ratio (WHR) was used to divide participants into low and high central adiposity groups with sex-specific median cut points (men 0.943, women 0.801). Six participants were excluded from the analysis because of incomplete data. All participants gave full informed consent and the study was approved by the University College London Hospitals Committee on the Ethics of Human Research.

### 2.2 Procedures

Questionnaires were completed before the mental stress testing sessions that were conducted individually in a light and temperature controlled laboratory in either the morning or the afternoon between 2006 and 2008. At the start of the session, anthropometric measurements were taken. Height was measured to the nearest 0.1 cm using a stadiometer and weight was measured in kilos (kg) using Tanita Scales. Body Mass Index (BMI) was subsequently calculated using this information (kg/m^2^). Waist circumference was assessed using a metal tape halfway between the bottom rib and iliac crest, and hip circumference was measured at the level of the great trochanters. The measurements were conducted twice to ensure accuracy, and WHR was calculated as waist circumference/hip circumference.

Following these measurements, the participant was seated and a venous cannula was inserted into the lower arm for the collection of blood samples. Systolic BP, diastolic BP and heart rate were assessed continuously using a Finometer (Finapres Medical Systems, Amsterdam). There followed a 30 min resting period, the last 5 min of which was used to measure baseline systolic and diastolic BP and heart rate. At this time the first blood sample was drawn into EDTA and citrated tubes, and a saliva sample was taken using Salivettes (Sarstedt, Leicester, UK).

Two 5-minute behavioural tasks that have been used to induce stress in a number of previous studies were then administered in a counterbalanced order (Steptoe et al., 2002b). These tasks have been shown to elicit consistent cardiovascular and inflammatory responses on repeat testing (Hamer et al., 2006). The first was a computerized version of the Stroop colour-word-interference task. Participants had to press a computer key to choose one out of four words that matched the colour of the target word presented at the centre of the screen. Target and distractor words were printed in incongruous colours (e.g. the word blue printed in red, etc.). The second task was mirror tracing, involving the tracing of a star with a metal stylus which could only be seen in mirror image. The device emitted a loud beep with every error. Participants were told that people usually manage to finish five circuits of the star during the allocated time.

A second blood sample and a saliva sample were taken immediately after the tasks, while systolic and diastolic BP and heart rate were measured continuously throughout the tasks and recovery period. Further blood samples were obtained 45 min and 75 min after task completion, with saliva samples at 20, 45 and 75 min during the recovery period.

Following the mental stress testing session, participants were asked to take 5 saliva samples over the next day, following methods previously used in this laboratory (Kunz-Ebrecht et al., 2004). Samples were requested on waking, 30 min later, at 10:00 h, 16:00 h and 20:00 h, and were sent back to the research office and stored at −20 °C.

### 2.3 Blood and salivary measures

Blood samples were centrifuged immediately at 2500 rpm for 10 min at room temperature and the separated plasma was aliquoted into 0.5 ml portions stored at −80 °C until further analysis. Three inflammatory markers were analysed. Plasma IL-6 was assayed using a Quantikine high sensitivity two-site enzyme-linked immunosorbent assay (ELISA) from R&D Systems (Oxford, UK). The sensitivity of the assay ranged from 0.016 to 0.110 pg/ml and the intra and inter assay CVs were 7.3% and 7.7% respectively. Fibrinogen was measured from citrated blood with an automated Clauss assay in a MDA-180 coagulometer (Oragon Teknika, Cambridge, UK) using the manufacturer's reagents and the International Fibrinogen Standard, with inter- and intra-individual CVs of 8%. High sensitivity C-reactive protein was assayed in duplicate with a high sensitivity enzyme immunoassay kit (BioCheck, CA, USA). Glycated haemoglobin (HbA1c) was measured using a Tosoh G7 high performance liquid chromatography analyser (San Francisco, CA) calibrated to Diabetes Control and Complications Trial standards. CMV IgG antibody titres were measured from thawed serum samples using a solid-phase enzyme immunoassay system as described previously (Dowd et al., 2013). A sample was categorized as seropositive if the optical density ratio was 1.0 or greater as read by a spectrophotometer. All CMV assays were carried out by the Stanley Neurovirology Laboratory of the Johns Hopkins University School of Medicine. Saliva samples were stored at −20 °C until analysis at the University of Dresden. Cortisol levels were assessed from saliva using a time resolved immunoassay with fluorescence detection, and the intra- and inter-assay coefficients of variation (CVs) were less than 8%.

### 2.4 Statistical analyses

Sociodemographic factors were compared across SES and WHR groups using analysis of variance for continuous and Chi^2^ tests for categorical variables. Cardiovascular data were averaged over 4 periods: the last 5 min of the baseline period (baseline), the two behavioural task trials, and 40–45 min and 70–75 min following tasks (recovery phase). Heart rate variability was modelled as root mean square of successive differences (RMSSD) over the same time periods, and was log transformed before analysis because of negative skew. The total cortisol output over the stress session was computed using the area under the curve (AUC) method (Pruessner et al., 2003), and a similar method was used to quantify cortisol output over the day. Cortisol over the day was negatively skewed so log transformed before analysis, but raw values are shown in Section 3. There were 41 women with current or recent hormone replacement treatment, but including this factor into account did not modify the results, so it was not included in the final models.

Baseline values of biomarkers were analysed by analysis of covariance with SES (grade of employment) and central adiposity (WHR) group as between-person factors, covarying for age, sex, and smoking status, since these potentially influence results. Analyses of salivary cortisol also included time of day of laboratory session (morning/afternoon) as a covariate. In order to identify effects related specifically to central adiposity, BMI was added as a further covariate to all analyses. Linear contrasts tested gradients across SES groups. The number of participants included in each analysis differed because of missing data, so the sample sizes are indicated in Section 3.

Stress responses were analysed using repeated measures analysis of variance with SES and WHR as between-person factors and trial as the within-person factor. There were 4 trials in the cardiovascular and IL-6 analyses: baseline, during task for cardiovascular variables and immediately after tasks for IL-6, and 45 and 75 min after tasks. Fibrinogen was sampled at 3 time points since levels return to baseline promptly after stress: baseline, immediately after tasks, and at 45 min (Steptoe et al., 2003b). Salivary cortisol was analysed across 5 trials (baseline, immediately after tasks, and 20, 45 and 75 min after tasks), and also by analysing the AUC for the session. The Greenhouse–Geisser correction of degrees of freedom was applied where appropriate. Stress reactivity was further analysed with difference scores between baseline and stress tasks, while post-stress recovery in cardiovascular variables was assessed as differences between task and recovery trials, with higher values indicating greater recovery.

C-reactive protein and HbA1c were measured once, and were analysed in the same fashion as the baseline values of biomarkers. We analysed CMV serostatus using logistic regression, and present the odds of CMV positivity (with 95% confidence intervals, CI) adjusted for age, sex, smoking status and BMI, with the higher SES / small WHR group as the reference category.

We also carried out two sensitivity analyses. First, we investigated the impact of WHR modelled as a continuously distributed as opposed to a categorical variable. Because of sex differences in WHR, normalised scores were used in these analyses. Second, we reanalysed all data with only SES and WHR in the models, without covarying for age, sex, smoking status, and BMI.

## 3 Results

There were 207 higher, 209 intermediate and 121 lower SES participants as defined by occupational grade (Table 1). There was a significant difference in age across SES groups (F(2,531) = 4.23, *p* = 0.015, since the lower SES participants were slightly older on average. Lower SES participants were more likely to be unmarried (*p* = 0.007), while educational attainment was positively associated with SES, with a substantially greater number of higher than lower SES volunteers being educated to University level (*p* < 0.001). There was no difference in the proportion of participants in the small and large WHR categories across SES groups, and no difference in gender distribution. As might be expected, BMI, average waist circumference and average hip circumference were all greater in the large than small WHR groups (*p* < 0.001). Additionally, there was a non-linear SES difference in waist circumference (*p* = 0.007), with slightly larger waists on average in intermediate than higher or lower SES respondents. There was a moderate positive correlation between WHR and BMI (*r* = 0.41), but tests for multicollinearity (variance inflation factors and tolerance) were all within acceptable ranges. Measures of task appraisal showed no differences related to SES or WHR in the extent to which tasks were perceived as difficult, involving, or controllable (results not shown).

**Table 1:** Characteristics of the study sample.

	Socioeconomic status					
	Higher (N = 207)		Intermediate (N = 209)		Lower (N = 121)	
Waist-hip ratio grouping	Small	Large	Small	Large	Small	Large
N	107	100	109	100	52	69
Age (years)	62.70 (5.78)	61.81 (5.26)	62.57 (5.49)	62.94 (5.56)	63.31 (5.71)	64.94 (6.11)
Sex (% men)	55 (51.4%)	59 (59.0%)	65 (59.6%)	58 (47.2%)	23 (44.2%)	28 (40.6%)
Marital status: unmarried (%)	38 (35.5%)	29 (29.3%)	35 (32.1%)	32 (32.0)	22 (42.3%)	35 (50.7%)
Education to University degree level (%)	61 (57.5%)	68 (68.7%)	30 (28.0%)	18 (18.8%)	4 (8.5%)	5 (8.2%)
Current smokers (%)	4 (3.7%)	6 (6.0%)	2 (1.8%)	10 (10.0%)	5 (9.6%)	3 (4.3%)
BMI (kg/m^2^)	23.97 (2.84)	27.55 (4.36)	24.72 (3.66)	27.32 (3.65)	23.94 (3.41)	27.51 (3.46)
Waist circumference (cm)	78.26 (9.35)	95.29 (11.03)	81.46 (10.93)	94.70 (10.68)	77.15 (9.41)	91.50 (9.93)
Hip circumference (cm)	95.6 (6.24)	101.2 (9.41)	97.7 (7.29)	100.2 (7.86)	95.1 (7.57)	100.6 (8.25)

Values are presented as Means (SD) and N (%).

### 3.1 Resting differences in biological activity

Baseline levels of cardiovascular, inflammatory and other biological measures are summarized in Table 2, adjusted for age, sex, smoking status and BMI. There were significant SES gradients in systolic and diastolic BP (F(2,512) = 10.28 and 7.47, *p* < 0.001), with lower BP in higher status participants. There were no SES differences in heart rate or heart rate variability at baseline. Systolic BP was not related to WHR, but both diastolic BP and heart rate were higher in the large WHR category after adjusting for covariates (F(1,512) = 5.83, *p* = 0.016 and F (1,469) = 4.40, *p* = 0.037). Baseline plasma IL-6 (F(2,509) = 3.03), fibrinogen (F(2,506) = 4.83) and C-reactive protein (F(2,510) = 2.29) all showed significant gradients across SES groups (all *p* = 0.033 or greater), with higher levels of inflammation in lower SES groups after adjustment for covariates. There was an SES gradient in baseline salivary cortisol as well (F(2,512) = 3.67, *p* = 0.009), with a higher concentration in lower SES participants. There were no significant differences in HbA1c levels. The interaction between SES and WHR was significant for plasma fibrinogen, (F(2,506) = 9.46, *p* < 0.001), and C-reactive protein, (F(2,510) = 4.20, *p* = 0.016). In both cases, the highest values were observed in the lower SES/large WHR group.

**Table 2:** Baseline levels of biological measures.

	Socioeconomic status					
	Higher (207)		Intermediate (209)		Lower (121)	
Waist-hip ratio grouping	Small	Large	Small	Large	Small	Large
Systolic BP (mmHg) *N* = 522	122.28 (13.95)	124.76 (14.29)	124.44 (14.82)	127.36 (14.41)	132.22 (15.22)	129.96 (14.57)
Diastolic BP (mmHg) *N* = 520	71.53 (8.51)	74.17 (8.31)	73.41 (10.76)	75.54 (9.82)	76.13 (9.11)	78.08 (10.31)
Heart rate (bpm) *N* = 479	65.85 (9.20)	67.13 (9.15)	65.81 (9.28)	67.19 (8.88)	65.08 (7.21)	68.28 (9.61)
Heart rate variability (rms, ln) *N* = 446	3.05 (0.38)	3.09 (0.52)	3.08 (0.45)	3.04 (0.49)	3.07 (0.49)	3.02 (0.55)
Plasma IL-6 (pg/ml) *N* = 519	1.19 (0.74)	1.28 (0.74)	1.43 (1.02)	1.30 (0.71)	1.32 (0.57)	1.58 (0.93)
Plasma fibrinogen (g/L) *N* = 516	3.06 (0.52)	3.09 (0.53)	3.20 (0.64)	3.02 (0.61)	3.08 (0.53)	3.48 (0.71)
Plasma C-reactive protein (mg/L) *N* = 520	1.30 (1.24)	1.70 (2.21)	1.81 (2.34)	1.51 (1.76)	1.47 (1.18)	2.58 (3.69)
Salivary cortisol (ln, nmol/ml) *N* = 523	1.84 (0.46)	1.90 (0.46)	1.85 (0.50)	1.92 (0.44)	1.99 (0.47)	2.03 (0.42)
HbA1c (mmol/mol) *N* = 530	36.1 (3.40)	35.5 (5.04)	36.4 (3.56)	35.9 (4.83)	37.2 (10.30)	36.7 (4.15)

Values are presented as means (SD) and are adjusted for age, sex, smoking status and BMI. Salivary cortisol additionally adjusted for time of day of stress testing.

### 3.2 Cardiovascular responses to acute stress

**Fig. 1:**
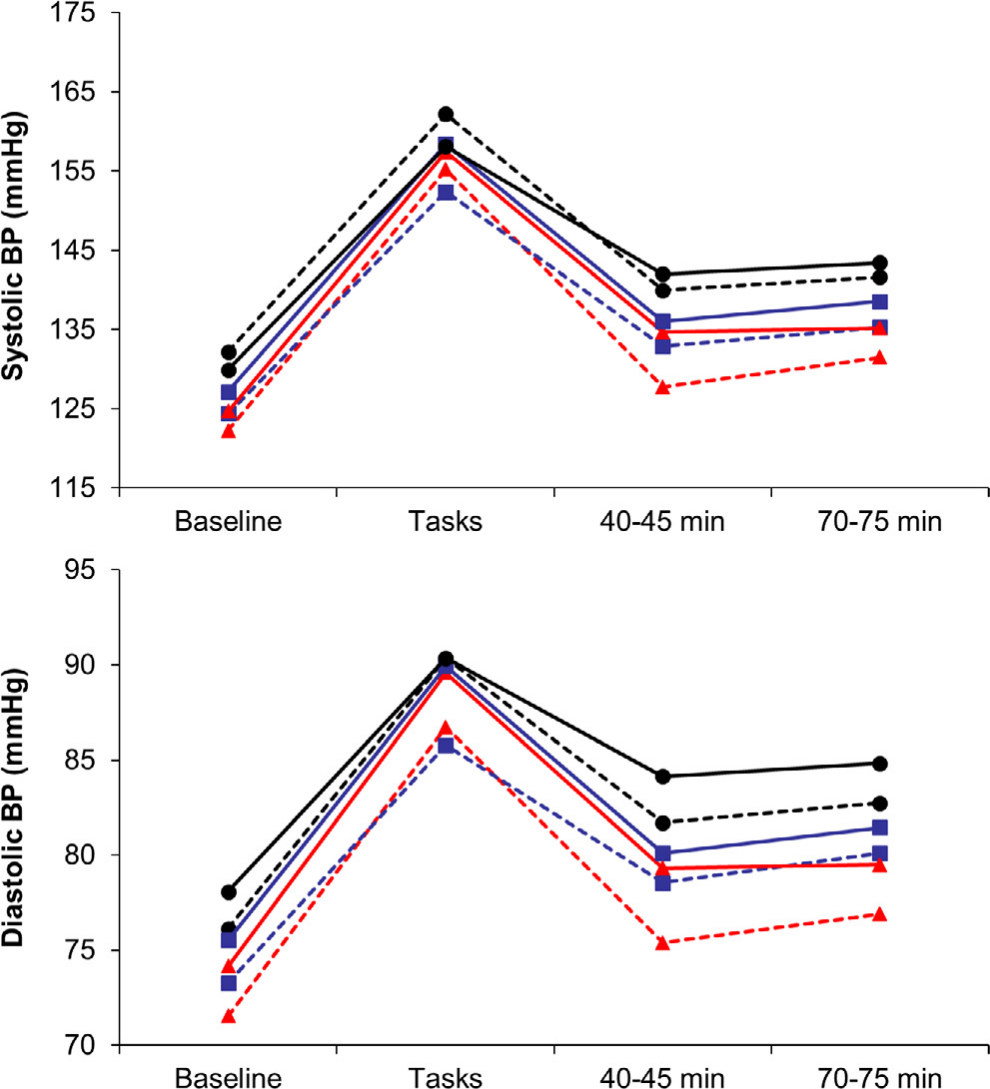
Mean levels of systolic and diastolic BP in relation to SES and WHR at baseline, during tasks, and 40–45 min and 70–75 min after task completion. Lower SES – black; Intermediate SES – blue; Higher SES – red; solid lines – large WHR; dashed lines – small WHR. Values are adjusted for age, sex, smoking status and BMI. Error bars are standard error of the mean (sem). (For interpretation of the references to colour in this figure legend, the reader is referred to the web version of this article.)

Repeated measures analysis of variance of systolic BP identified a main effect of SES (F(2,508) = 8.17, p < 0.001), together with an SES by trial interaction (F(6,1524) = 3.58, *p* = 0.005), and a 3-way SES by WHR by trial cubic effect (F(6,1524) = 3.24, *p* = 0.040). In the case of diastolic BP, there were main effects of WHR (F(2,507) = 5.73, *p* = 0.017) and SES (F(2,507) = 7.21, p < 0.001), plus significant SES by trial (F(6,1521) = 4.50, *p* < 0.001), and SES by WHR by trial cubic effects (F(2,507) = 3.36, *p* = 0.036). These results are summarised in Fig. 1, where it is apparent that BP increased markedly in response to mental stress, returning towards baseline during the recovery period. There was no difference in systolic BP reactions to tasks in relation to SES or WHR, but differences emerged during the recovery period. After adjustment for age, sex, smoking status and BMI, there was a significant SES gradient in the extent of recovery at 45 min (F(2,508) = 5.12, *p <* 0.001) and 75 min following stress (F(2,508) = 12.70, *p* < 0.001). SES effects remained significant after additional control for baseline and levels during tasks (F(2,505) = 3.57 and 3.93, *p* = 0.008 and 0.011 respectively), so were not secondary to any differences in baseline or stress reactivity. Additionally, SES and WHR interacted in recovery at 45 min (F(2,508) = 4.01, *p* = 0.019). These results are summarized in Table 3, and show that the amount of impaired recovery in the lower SES group was accentuated in the large WHR category. A similar pattern emerged for diastolic BP, with SES differences at 45 and 75 min (F(2,507) = 6.01 and 7.07, *p* = 0.003), with a SES and WHR interaction at 45 min showing that central adiposity moderated these SES differences (F(2,507) = 4.03, *p* = 0.018). As in the case of systolic BP, SES differences in recovery remained significant following additional adjustment for baseline and task levels at 45 min (F(2,504) = 4.57, *p* = 0.004) and 75 min (F(2,504) = 6.94, *p* = 0.002).

**Table 3:** Cardiovascular recovery following stress.

	Socioeconomic status					
	Higher (207)		Intermediate (209)		Lower (121)	
Waist-hip ratio grouping	Small	Large	Small	Large	Small	Large
*Systolic BP (mmHg)*						
Recovery at 45 min	26.60 (16.72)	22.46 (15.47)	19.45 (14.76)	22.38 (15.88)	22.15 (15.57)	16.22 (15.25)
Recovery at 75 min	24.51 (16.36)	21.94 (16.98)	18.98 (14.85)	19.15 (16.79)	18.12 (13.86)	14.09 (13.26)

*Diastolic BP (mmHg)*						
Recovery at 45 min	11.38 (7.14)	10.04 (7.12)	7.23 (7.55)	9.84 (8.78)	8.63 (6.39)	7.53 (7.03)
Recovery at 75 min	9.86 (6.85)	9.75 (8.45)	5.66 (7.29)	8.49 (8.18)	7.61 (6.76)	6.86 (7.00)
Heart rate (bpm)						
Recovery at 45 min	11.69 (7.59)	11.94 (7.59)	10.17 (7.19)	10.78 (5.43)	9.58 (5.17)	8.87 (7.48)
Recovery at 75 min	11.87 (7.65)	11.48 (7.92)	9.88 (7.17)	10.39 (5.50)	9.20 (6.17)	9.18 (7.19)

Values are presented as means (SD) and are adjusted for age, sex, smoking status and BMI.

**Fig. 2:**
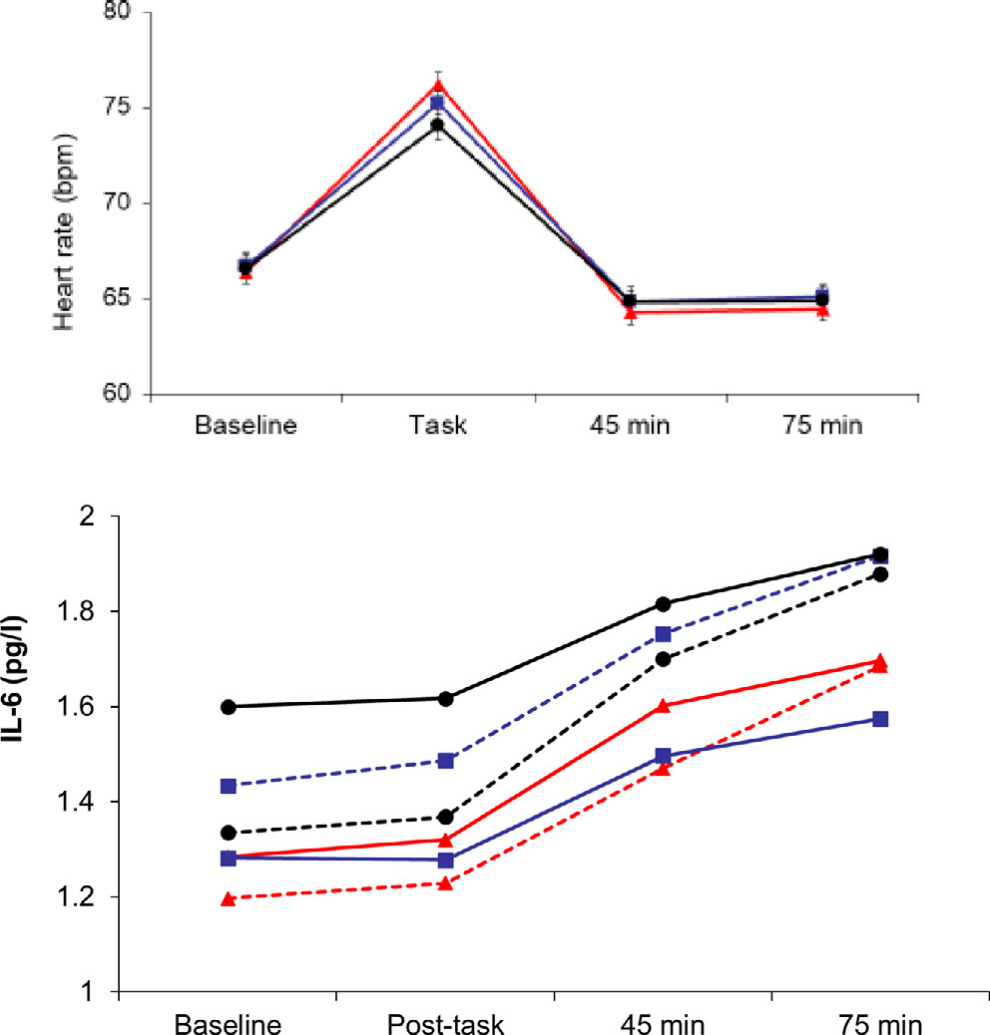
Mean levels of heart rate and plasma IL-6 during or immediately after tasks, and 40–45 min and 70–75 min after task completion. Heart rate results are for SES only, while IL-6 findings are plotted in relation to SES and WHR. For other details, see legend to Fig. 1.

The analysis of heart rate revealed an SES by trial interaction (F(6,1380) = 4.02, p < 0.001) and a main effect of WHR (F(1, 460) = 4.34, *p* = 0.034), but no 3-way interaction. In contrast with the BP results, there was a significant difference in heart rate reactions across SES groups (F(2,469) = 4.36, *p* = 0.004), with larger increases in the higher SES compared with lower SES participants (see Fig. 2). However, SES differences in recovery were the same as for BP at 45 min and 75 min (F(2,463) = 4.86 and 4.59, *p* = 0.004), with more effective recovery among higher SES participants (Table 3). These recovery effects remained significant with additional control for baseline levels. Heart rate variability also changed in response to the stress protocol (F(3,1314) = 231.5, *p* < 0.001), falling from baseline during tasks, and recovering above baseline after tasks. This pattern did not vary with SES, but there was a significant WHR by trial interaction (F(3,1302) = 2.81, *p* = 0.048). Responses to tasks were comparable, but the large WHR group showed better post-stress recovery than the small WHR group at 45 min (*p* = 0.037).

### 3.3 Inflammatory and neuroendocrine responses to acute stress

**Fig. 3:**
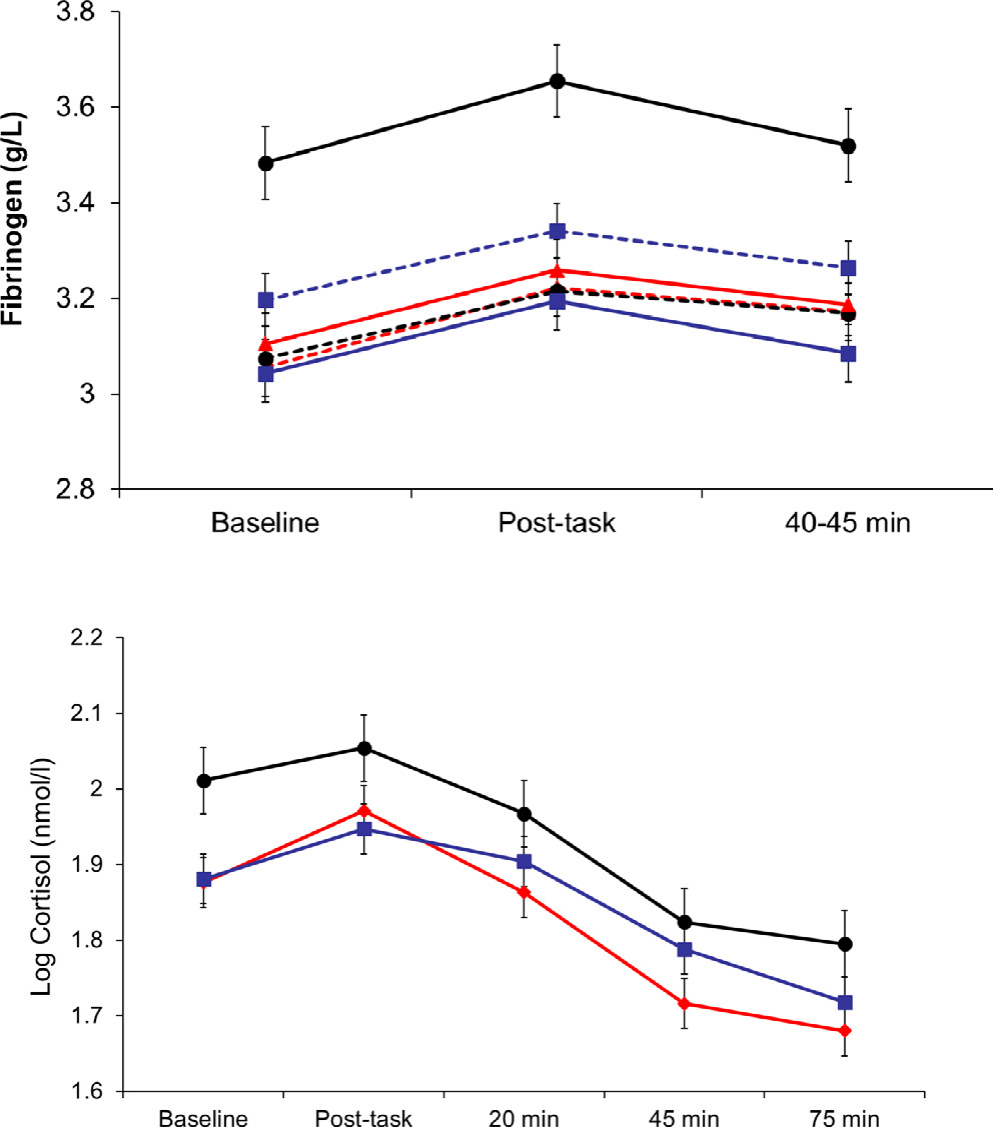
Mean levels of plasma fibrinogen and cortisol at baseline, immediately after tasks, and at 20 min (cortisol), 45 min and 75 min after task completion. Fibrinogen results are for SES and WHR, while cortisol is plotted in relation to SES only. For other details, see legend to Fig. 1.

Plasma IL-6 increased following stress, showing a delayed response with raised levels 45 and 75 min after tasks (F(3,1491) = 125.5, *p* < 0.001). There was also a significant SES effect (F(2,493) = 2.30, *p* = 0.033), with higher IL-6 concentrations in lower SES participants after adjustment for age, sex, smoking status and BMI (Fig. 2). The acute inflammatory responses to stress did not differ across SES groups, but the highest absolute levels were recorded in lower SES participants following stress. Additionally, the WHR group by trial interaction was significant (F(1,493) = 4.74, *p* = 0.030). The large WHR group showed smaller increases in IL-6 concentration over the session after adjustment for covariates, but there was no interaction between SES and WHR. The pattern of plasma fibrinogen responses differed from that for IL-6, in that increases with tasks were immediate and transient (F(2,992) = 4.99, *p* = 0.007). There was a significant main effect for SES, together with an SES by WHR interaction (F(2,496) = 8.29, *p* < 0.001). As can be seen in Fig. 3, this was primarily attributable to the substantially higher concentrations of fibrinogen in the lower SES/large WHR group.

The salivary cortisol AUC showed a main significant effect of SES (F(2,512) = 2.10, *p* = 0.041, mirroring the socioeconomic gradient at baseline described in Table 2. Additionally, analysis of individual samples identified an SES by trial interaction illustrated in Fig. 3 (F(2,512) = 4.13, *p* = 0.017). The elevated cortisol in the lower SES group is apparent throughout the session, but there is no clear relationship with stress responsivity or recovery.

### 3.4 CMV serostatus, SES and central adiposity

A substantial proportion of respondents were positive for CMV, indicating that past infection was relatively common. Table 4 summarises the results in relation to SES and WHR. Logistic regression revealed that the odds of CMV seropositivity were increased more than twofold in the lower SES/large WHR group compared with the higher SES/small WHR group after adjustment for covariates (*p* = 0.009). The prevalence of positive CMV serostatus was 44.8% in the higher SES/small WHR group compared with 66.0% in the lower SES/large WHR group.

**Table 4:** CMV serostatus, SES and central adiposity.

Group	Percent (95% CI)	Adjusted odds ratio (95% CI)	p
Higher SES/small WHR	44.8% (34.8–54.7)	Reference (1)	
Higher SES/large WHR	47.2% (36.9–57.4)	1.10 (0.61–2.00)	0.75
Intermediate SES/small WHR	48.2% (38.5–57.8)	1.15 (0.66–2.00)	0.62
Intermediate SES/large WHR	44.5% (34.5–54.5)	0.98 (0.54–1.78)	0.96
Lower SES/small WHR	48.1% (34.2–62.0)	1.14 (0.57–2.29)	0.71
Lower SES/large WHR	66.0% (53.8–78.3)	2.47 (1.35–4.89)	0.009

Adjusted for age, sex, smoking status and BMI. N = 511.

SES = socioeconomic status; WHR = waist-hip ratio

### 3.5 Cortisol across the day

**Fig. 4:**
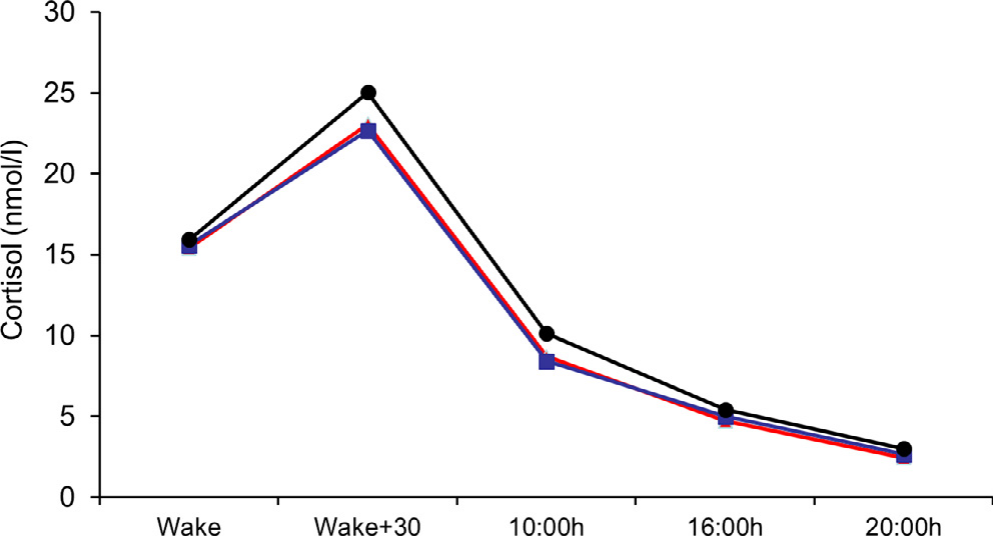
Mean salivary cortisol concentration across the day in relation to SES. For other details, see legend to Fig. 1.

There was a typical salivary cortisol profile across the day, with high values on waking and an increase in cortisol 30 min later, followed by progressive decline into the evening. Significant differences by SES were apparent (F(2,458) = 4.93, *p* = 0.006), with greater cortisol output among lower SES individuals (Fig. 4). There was, however, no interaction between SES and time of day, and no SES difference in the rise in cortisol between waking and 30 min later, or in the slope of cortisol decline across the day. Cortisol output over the day was not related to WHR, and there were no significant interactions between SES and WHR. Analyses of men and women separately showed no differences in these patterns of change over the day (results not shown).

### 3.6 Sensitivity analyses

The first sensitivity analysis investigated whether associations would be different if WHR modelled as a continuous variable rather than categorising individuals as having larger or smaller WHR. The results, summarised in Appendix A, Table 1, indicate very few differences from the categorical analyses. Three interactions between SES and WHR were no longer significant with continuous WHR, but there were no differences from the main analysis in baseline or resting effects for systolic or diastolic BP, heart rate, HRV, IL-6, salivary cortisol or CMV serostatus, and no differences from the main analysis in reactivity or recovery effects for any outcome apart from diastolic BP. The pattern of results for salivary cortisol over the day was unchanged.

The second sensitivity analysis assessed the associations of SES and WHR with biology in the absence of statistical adjustment for age, sex, smoking status and BMI. The results are summarised in Appendix A, Table 2. Significant associations between WHR and resting HRV and plasma IL-6 emerged that were not present in the main analysis. In relation to systolic BP, the interaction between WHR and trial was significant (this was conditional on SES in the main analysis), and there was a main effect of WHR on plasma IL-6, with higher IL-6 in the larger than smaller WHR group throughout the session. By contrast, the previously observed WHR by trial interaction was no longer significant. These effects primarily emerged because of the absence of BMI as a covariate. Otherwise, there were no differences from the main analyses in baseline levels, stress reactivity and recovery, or in cortisol output over the day.

## 4 Discussion

This study investigated the separate and combined associations between lower SES and central adiposity and a range of stress-related biological processes. We hypothesised that lower SES and greater central adiposity (waist-hip ratio) would be associated with impaired post-stress recovery in cardiovascular parameters, with heightened inflammatory and neuroendocrine responses to stress, and with greater cortisol output over the day. We also conjectured that the combination of lower SES and greater central adiposity would be particularly toxic. The results confirmed and extended previous studies of SES and cardiovascular, inflammatory and neuroendocrine factors, and identified important interactions between lower SES and central adiposity in relation to inflammatory markers, CMV infection, and post-stress recovery of cardiovascular function.

Associations between SES and acute cardiovascular stress responsivity have been investigated in a number of studies. A meta-analysis in 2018 showed no consistent relationship between SES and BP or heart rate reactivity, but differences emerged in post-stress recovery, albeit in a sample of only 6 studies (Boylan et al., 2018). The present results are consistent with these conclusions and with a previous study from our laboratory (Steptoe et al., 2002a), in showing impaired systolic and diastolic BP and heart rates recovery following stress in lower SES participants. Heart rate variability did not follow this pattern, but was only measured using RMSSD, and frequency-based parameters may have been more sensitive. The study population was purposively recruited using a stratified sampling procedure to ensure adequate representation of people from different SES groups who were in good cardiometabolic health, assuaging some of the heterogeneity in sampling present in some other studies. Interestingly, we observed higher heart rate reactivity to tasks in the higher SES group. A similar pattern has been reported previously, and may relate to the performance of cognitive tasks as opposed to other types of behavioural challenge (de Rooij, 2013). We selected the tasks in this study after pilot work on a number of alternatives, choosing those that were appraised similarly by people across the SES spectrum (Steptoe and Marmot, 2002), and there were no differences in task appraisal in the present study.

Although central adiposity did not affect cardiovascular reactivity or recovery directly, we observed interactions between SES and WHR in the post-stress recovery profile of systolic and diastolic BP. Blood pressure recovery was markedly impaired in the lower SES /large WHR group. For example, the decrease in systolic BP from task levels at 45 min averaged 26.6 mmHg in the higher SES/small WHR group, 22.15 mmHg in the lower SES/small WHR group, but only 16.22 mmHg in the lower SES/large WHR group (Table 3). Impaired post-stress recovery has previously been shown to predict clinical hypertension and cardiovascular disease risk (Panaite et al., 2015). Our results suggest that this pathway may link greater central adiposity with cardiovascular risk among lower SES adults.

Resting values of all three inflammatory markers in this study (C-reactive protein, IL-6 and fibrinogen) were inversely associated with SES in a graded fashion (Table 2). This relationship has been recorded in larger epidemiological studies as well (Nazmi and Victora, 2007, Steptoe, 2012), and suggests that lower SES is characterised by low grade systemic inflammation that may contribute to increased risk for several physical and mental health outcomes, particularly at older ages (Franceschi et al., 2018). The lower SES participants with greater central adiposity had particularly raised levels of resting C-reactive protein and plasma fibrinogen compared with other groups, together with sustained elevations in fibrinogen throughout the mental stress protocol. Inflammatory processes play a key role in linking cardiovascular disease with fat distribution (Ferrucci and Fabbri, 2018). Central or abdominal fat stores are a potent source of adipokines while also contributing to high circulating levels of very-low-density lipoprotein, reduced high-density lipoprotein concentrations, and excess fatty acid flux (Piche et al., 2018). Central adiposity predicts cardiovascular risk independently of total body fat or BMI (Abraham et al., 2015, Liu et al., 2010). Plasma IL-6 did not conform to this pattern. The SES gradients in IL-6 and fibrinogen present at baseline were sustained throughout the study, but the increases in IL-6 concentration following stress occurred in parallel across the three SES groups. So although the highest levels were apparent in the lower SES group at 75 min post-stress, there was no differential reactivity. In a previous study, we observed that IL-6 concentration continued to increase among lower SES participants between 75 and 120 min following stress, whereas it stabilised in the higher SES group (Brydon et al., 2004). It is possible therefore that differences might have emerged if blood sampling had continued. Fibrinogen responses were more transient, but again occurred in parallel across SES groups, replicating results in an earlier smaller study (Steptoe et al., 2003a). There was a flatter rise in IL-6 among people with greater WHR, but no interaction with SES.

The cortisol responses to this protocol were small, although individual differences still predicted variations in cardiovascular risk and telomere attrition longitudinally (Hamer et al., 2012, Steptoe et al., 2017). Salivary cortisol was elevated throughout the mental stress testing session in lower compared with intermediate and higher SES groups, and lower SES individuals also showed greater cortisol output over the day (Fig. 2, Fig. 4). There were SES differences in the changes in cortisol across the stress session, but it is difficult to interpret these as differences in rate of post-stress recovery (Le-Scherban et al., 2018). Studies relating SES with diurnal cortisol profiles have generated conflicting results, although several have documented flatter slopes of cortisol recline over the day (Desantis et al., 2015, Hajat et al., 2010). Others have focused on the cortisol awakening response (Kunz-Ebrecht et al., 2004). We did not find any differences in the cortisol awakening response or in slope of decline over the day, but instead a difference in overall output that was independent of age, sex, smoking, and BMI.

The absence of differences in cortisol responses between WHR groups was unexpected, since several previous studies have shown associations (Bjorntorp, 2001, Incollingo Rodriguez et al., 2015). It is notable that the majority of studies in this field have assessed associations of cortisol with central adiposity without adjusting for BMI as was done in these analyses (Champaneri et al., 2013, Incollingo Rodriguez et al., 2015). As can be seen in Appendix A, Table 2, analyses that did not take general adiposity into account revealed more extensive differences related to WHR than did the adjusted analyses. But failure to adjust for BMI makes it more difficult to identify specific associations with central adiposity. Another explanation for the lack of striking associations with central obesity may relate to the study sample. Although the differences in central adiposity between the small and large WHR groups were marked, the body weights of this healthy cohort of older men and women were generally relatively moderate. It is possible that stress-related biological and psychosocial processes implicated in central adiposity will only be manifest among more overweight or obese individuals.

The interaction between lower SES and greater central adiposity in the proportion of respondents with CMV infection has not been documented before (Table 4). A socioeconomic gradient in CMV infection has also been noted in population studies (Dowd et al., 2009), and positive serostatus predicts all-cause mortality, CHD and hypertension risk (Du et al., 2018, Hui et al., 2016, Simanek et al., 2011). Molecular biological studies suggest that the infection is concentrated in endothelial cells leading to endothelial injury, subsequently affecting smooth muscle cell proliferation and promoting atherogenesis (Du et al., 2018). The present study cannot determine whether lower SES individuals with greater abdominal adiposity are at higher risk of infection, or whether CMV infection among lower SES groups drives the development of central adiposity. Nonetheless, the interaction suggests that infectious pathways to cardiovascular disease risk may be particularly important among less affluent people with central adiposity.

This study was carried out with a well characterised population that was selectively sampled to encompass a broad range of SES. Existing cardiovascular disease was excluded, so that associations are not secondary to differences in health status. The size of the sample was large by comparison with many investigations of stress-related inflammatory responses. However, the selection process resulted in a sample of individuals with relatively normal body weights, and only 14% had a BMI ≥ 30. The impact of central adiposity may be greater among people who have more general obesity. The study was carried out with men and women of white European origin, and results may not generalise to other groups. SES was defined by occupational status, and other criteria such as educational attainment or income might generate different results. The cross-sectional design makes it impossible to draw causal conclusions about the directionality of effects. It is likely that there is a bidirectional relationship between central adiposity and stress biology, with greater abdominal fat contributing to the expression of adipokines and other markers, while stress stimulates more rapid accumulation of fat stores. Findings may also be limited by having only one session of mental stress testing and sampling cortisol over a single day. Nonetheless, the findings extend the evidence relating lower SES to stress-related biological risk factors for cardiovascular disease, while indicating that central adiposity may augment SES differences in inflammation and impaired recovery in cardiovascular function following acute mental stress.

## Conflicts of interest

The authors have no conflicts of interest to declare.

## Funding

This research was supported by the British Heart Foundation (RG/10/005/28296).
