# Supplementary material for: Socioeconomic status and central adiposity as determinants of stress-related biological responses relevant to cardiovascular disease risk
Source: Brain Behav Immun. 2019 Mar;77:16–24. doi: 10.1016/j.bbi.2018.11.019 (PMC6417991; doi:10.1016/j.bbi.2018.11.019)
Supplement: Supplementary data 1 [file mmc1.pdf]

Socioeconomic status and central adiposity as determinants of stress-related biological responses relevant to cardiovascular disease risk

Supplementary table 1

Analyses based on continuously distributed waist-hip ratio

Variable	Differences from categorical analysis	Statistics<br>Standardised regression (β), standard error (SE), *p* value
Baseline / resting values		
Systolic BP	No difference	
Diastolic BP	No difference	
Heart rate	No difference	
Heart rate variability	No difference	
Plasma IL-6	No difference	
Plasma fibrinogen	SES by WHR not significant	β = 0.198, SE = 0.109, $p = 0.07$
C-reactive protein	SES by WHR not significant	β = 0.104, SE = 0.110, $p = 0.35$
Salivary cortisol	No difference	
HbA1c	No difference	
CMV serostatus	No difference	
Reactivity / recovery		
Systolic BP	No difference	
Diastolic BP	SES by WHR not significant	β = 0.012, SE = 0.092, $p = 0.89$
Heart rate	No difference	
Heart rate variability	No difference	
Plasma IL-6	No difference	
Plasma fibrinogen	No difference	
Salivary cortisol	No difference	
Values over the day		
Salivary cortisol	No difference	

Supplementary table 2

Unadjusted analyses of SES and WHR

Variable	Differences from categorical analysis	Statistics<br>F ratios and *p* values
Baseline / resting values		
Systolic BP	No difference	
Diastolic BP	No difference	
Heart rate	No difference	
Heart rate variability	Lower HRV in greater WHR group	F(1,440) = 4.58, $p = 0.033$
Plasma IL-6	Higher IL-6 in greater WHR group	F(1,513) = 11.56, $p = 0.001$
Plasma fibrinogen	No difference	
C-reactive protein	No difference	
Salivary cortisol	No difference	
HbA1c	No difference	
CMV serostatus	No difference	
Reactivity / recovery		
Systolic BP	WHR by trial interaction, with poorer recovery in higher WHR group	F(3, 1536) = 3.21, $p = 0.036$
Diastolic BP	No difference	
Heart rate	No difference	
Heart rate variability	No difference	
Plasma IL-6	Main effect of WHR, but no WHR by trial interaction	F(1,497) = 7.66, $p = 0.006$
Plasma fibrinogen	No difference	
Salivary cortisol	No difference	
Values over the day		
Salivary cortisol	No difference	
